# PET/CT Imaging of Infectious Diseases: Overview of Novel Radiopharmaceuticals

**DOI:** 10.3390/diagnostics14101043

**Published:** 2024-05-17

**Authors:** Ferdinando F. Calabria, Giuliana Guadagnino, Andrea Cimini, Mario Leporace

**Affiliations:** 1 Department of Nuclear Medicine and Theragnostics, “Mariano Santo” Hospital, 87100 Cosenza, Italy; m.leporace@aocs.it; 2 Department of Infectious and Tropical Diseases, St. Annunziata Hospital, 87100 Cosenza, Italy; 3 Nuclear Medicine Unit, St Salvatore Hospital, 67100 L’Aquila, Italy; andreacimini86@yahoo.it

**Keywords:** PET/CT, ^68^Ga-citrate, ^18^F-FDG, PET/MRI, infection, inflammation, molecular imaging

## Abstract

Infectious diseases represent one of the most common causes of hospital admission worldwide. The diagnostic work-up requires a complex clinical approach, including laboratory data, CT and MRI, other imaging tools, and microbiologic cultures. PET/CT with ^18^F-FDG can support the clinical diagnosis, allowing visualization of increased glucose metabolism in activated macrophages and monocytes; this tracer presents limits in differentiating between aseptic inflammation and infection. Novel PET radiopharmaceuticals have been developed to overcome these limits; ^11^C/^18^F-labeled bacterial agents, several ^68^Ga-labeled molecules, and white blood cells labeled with ^18^F-FDG are emerging PET tracers under study, showing interesting preliminary results. The best choice among these tracers can be unclear. This overview aims to discuss the most common diagnostic applications of ^18^F-FDG PET/CT in infectious diseases and, as a counterpoint, to describe and debate the advantages and peculiarities of the latest PET radiopharmaceuticals in the field of infectious diseases, which will probably improve the diagnosis and prognostic stratification of patients with active infectious diseases.

## 1. Introduction

Infectious diseases represent one of the most common causes of hospital admission in the world [1]. Usually, the diagnosis is reached by a clinical approach, including laboratory data, *X*-ray, other imaging tools, and microbiologic cultures. However, in some patients, the infectious process cannot be adequately identified with conventional imaging; in this clinical setting, nuclear medicine can be of help, allowing the identification of infectious foci with *Single Photon Emission Computed Tomography* (SPECT) and/or *Positron Emission Tomography* (PET); both scans allow the precise identification of infectious diseases and permit the assessment of response to therapy, with diagnostic accuracy superior to that of CT and MR imaging [2].

Moreover, the co-registration with CT, generally performed as a *low-dose* scan, allows the adequate depiction of localization and extension of the infectious focus, the involvement of surrounding tissues (i.e., *the skeleton*), and possible distant septic emboli [3,4].

Due to the worldwide distribution of hybrid scanners, PET/CT imaging of infectious diseases has been proven to be useful in the management of such patients, with the added value of 2-deoxy-2-[^18^F]-fluoro-D-glucose (^18^F-FDG). Being an analogue of glucose, ^18^F-FDG is taken up in inflammatory cells actively participating in the immune-inflammatory cascade, such as monocytes, macrophages and neutrophils [5], due to the local up-regulation of glucose transporters caused by inflammatory mediators, leading to high intracellular ^18^F-FDG accumulation.

Therefore, ^18^F-FDG PET/CT imaging is currently useful in the management of patients with systemic diseases or regional infectious diseases such as infection of vascular grafts [6], prosthetic joints [7], and device-related [8] infections, *Fever of Unknown Origin* (FUO) [9], granulomatosis [10], and endocarditis [11].

More recently, after the COVID-19 pandemic [12], ^18^F-FDG PET has also been hypothesized to have a feasible role in the detection and assessment of responses related to COVID-19 disease activity, holding significant clinical relevance due to the extreme versatility of ^18^F-FDG for its intrinsic molecular properties [13]. Beyond all of these cited features, as already known from oncologic studies, ^18^F-FDG PET suffers from a lack of specificity.

Both infectious and inflammatory diseases comprise a large number of different pathologies with heterogeneous clinical presentations, potentially confined to a precise district or with systemic involvement. ^18^F-FDG PET/CT is a very sensitive whole-body tool based on the relatively non-specific tracer uptake; moreover, in comparison with SPECT imaging with radiolabeled leukocytes, it is more rapid, with higher image resolution, and does not imply handling of blood. Nevertheless, this feature also represents the main limit of its use; in fact, the main advantage of ^18^F-FDG is also the basis for its diagnostic pitfalls and limitations. Its low specificity can hinder the differentiation between pathologic and physiologic uptake, particularly in the brain, liver, myocardium, and bladder [14]. Furthermore, the differentiation between aseptic inflammation and infectious foci can be difficult.

More specific PET radiopharmaceuticals, labeled with ^18^F and other nuclides such as ^11^C and ^68^Ga [15,16], are currently under study. These novel tracers have already shown promising features for the evaluation of systemic and/or localized infectious foci.

Our overview aims to discuss the most common diagnostic applications of ^18^F-FDG PET/CT in infectious diseases and, as a counterpoint, to describe and debate the latest radiopharmaceuticals in the field of hybrid PET/CT imaging of infectious diseases, which will probably improve the diagnosis and prognostic stratification of patients with active infectious diseases.

## 2. ^18^F-FDG in Infectious Diseases

**Figure 1:**
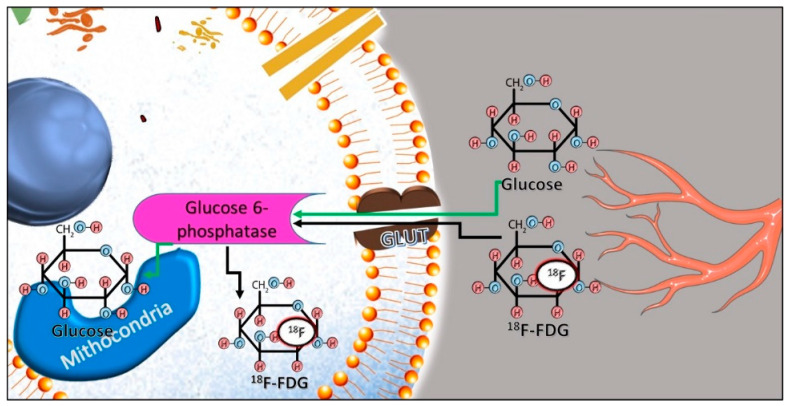
Internalization and phosphorylation of ^18^F-FDG from the bloodstream in cells.

The ^18^F-FDG uptake in infectious and inflammatory conditions follows the same metabolic pathway known for malignant lesions. The elevated rate of glucose metabolism in cells is mediated by the upregulation of the glucose transporter (GLUT), also representing the basis for the increased ^18^F-FDG uptake in monocytes, macrophages and neutrophils, in comparison to normal cells. After internalization in the cytoplasm, glucose is phosphorylated by the enzyme hexokinase to facilitate further processing through the glycolytic pathway, while ^18^F-FDG undergoes the same process. Due to the chemical differences, ^18^F-FDG-6-phosphate is not a substrate for the downstream enzymes in the glycolytic pathway [14], the process is not advanced further, and the tracer remains trapped inside the cell (Figure 1).

### 2.1. Prosthesis Joint Infection

Since ^18^F-FDG uptake persists in prosthesis joint infection cases, PET/CT can help in differentiating aseptic loosening from infection. Several studies demonstrated that ^18^F-FDG PET/CT shows good diagnostic accuracy in the detection of prosthesis infection, with high sensitivity and specificity [17,18]. This is because activated macrophages continue to accumulate ^18^F-FDG in chronic infection.

In particular, PET/CT imaging with ^18^F-FDG can provide additional information over conventional modalities by adding information on the disease extent in the bone as well as in the whole body, giving an idea of active pathology at the molecular level, and in the evaluation of response to therapy [19].

Therefore, some authors support its usefulness in the diagnosis and monitoring of infection in hip and knee prostheses, with a diagnostic accuracy superior to that of scintigraphy with labeled leukocytes [20,21]. On the other hand, it is important to state that a lack of specificity must be kept in consideration when examining patients with chronic prosthetic joint infections with ^18^F-FDG PET/CT; for this reason, authors from a multidisciplinary panel suggest that accurate diagnosis can be prospectively obtained with a diagnostic algorithm including multi-modal radionuclide imaging, involving ^99^Tc-phosphate SPECT/CT, ^111^In-labeled leukocytes and ^99^Tc-nanocoll bone marrow SPECT/CT, and ^18^F-FDG PET/CT. This approach could overcome the limits of all techniques before submitting patients to revision surgery of an infected prosthesis after total hip or knee arthroplasty [22].

### 2.2. Pulmonary Granulomatosis

Sarcoidosis is an idiopathic, granulomatous non-caseating disease generally involving the lungs and mediastinal lymph nodes but potentially affecting all organs. The clinical outcome is variable, with a significant percentage of patients needing systemic treatment [23]. PET/CT with ^18^F-FDG enables tracer uptake in granulomatous cells producing the inflammation seen in sarcoidosis, with good sensitivity in the detection of mediastinal and extra-thoracic lesions [24]. On this basis, hybrid PET/CT imaging is a valid method in the diagnosis and monitoring of response to therapy in patients with histologically proven sarcoidosis [25].

Tuberculosis is another granulomatous disease characterized by caseating lesions, still representing the leading cause of death from an infectious disease worldwide, among *human-immunodeficiency virus* (HIV) negative people and HIV-positive patients [26]. Mycobacterium tuberculosis remains one of the most lethal human pathogens since drug-resistant tuberculosis and HIV-related tuberculosis infection represent diagnostic and therapeutic challenges. Considering that tuberculous granulomatous inflammation appears as ^18^F-FDG avid lesions on PET/CT scans [27], this tool enables the delineation of disease extent and assessment of occult extra-pulmonary lesions in whole-body imaging [28]. Furthermore, ^18^F-FDG PET/CT imaging is also effective in assessing treatment response during and after the therapy [28,29]. ^18^F-FDG PET/CT imaging may be also useful in schistosomiasis, especially in the identification of infection sites: schistosomiasis is one of the most important parasitic infections in tropical and subtropical areas and represents a further cause of granulomatous disease, in which schistosome eggs trapped in organs lead to severe inflammatory reactions, including granulomas, appearing hypermetabolic on ^18^F-FDG PET/CT [30].

### 2.3. Vasculitis

^18^F-FDG PET/CT is an important component of the diagnostic work-up required for patients with large vessel vasculitis, such as Takayasu arteritis and giant cell arteritis. These diseases are vasculitis involving the aorta and/or large arteries. The presence of diffusely increased ^18^F-FDG activity along the aortic wall and its major branches may enable the diagnosis of patients with clinical signs and symptoms of vascular inflammation, with good sensitivity and specificity for Takayasu arteritis [31,32]. A late ^18^F-FDG PET-CT (performed during the first 10 days from the beginning of the treatment) can also help in assessing response to therapy [33]. Similar features are documented for giant cell arteritis [34], while its role is unclear in the management of smaller arteries, due to the low PET/CT power resolution limit.

### 2.4. Fever of Unknown Origin (FUO)

The diagnosis of fever of unknown origin (FUO) still represents a challenge, due to the complex clinical presentations of patients, also considering that the leading cause may be located anywhere throughout the body. ^18^F-FDG PET is involved in the *frontline* in the differential diagnosis of lymphoma or malignant neoplasms in patients with FUO and lymphadenopathies [35,36]. In this clinical setting, a whole-body PET/CT may suggest the diagnosis of a lymphoproliferative disorder by showing the pattern of distribution of tracer-avid lesions and displaying the best target for biopsy [37]. As reported above, ^18^F-FDG may also help in identifying the vasculitis potentially underlying the clinical onset in FUO [31].

Bacteriemia of unknown origin is a further condition linked to FUO, in which ^18^F-FDG PET may help the identification of the infectious focus, particularly in pediatric [38,39] or immunocompromised patients with acquired immune deficiency syndrome (AIDS) [40].

### 2.5. Endocarditis and Cardiac Electronic Device Infections

Early diagnosis of endocarditis is crucial for optimal patient management, due to its high rates of mortality and morbidity [11]. The diagnosis can be difficult, involving a multidisciplinary discussion in addition to the integration of clinical signs, microbiology, and imaging data. Among imaging tools, ^18^F-FDG PET/CT is a valid approach to assessing the state of tracer distribution in the heart, also excluding other septic emboli or localizations, being a whole-body imaging tool. Thus, the European guidelines for the management of infective endocarditis have indeed incorporated intra-cardiac findings from ^18^F-FDG-PET/CT as major criteria for infective endocarditis [41].

The sensitivity and specificity of ^18^F-FDG PET/CT are respectively 86% and 84% [42,43]. Among other advantages, ^18^F-FDG PET enables the identification of possible portals of entry or the alternate diagnosis of infectious or inflammatory syndromes other than endocarditis. However, the high gradient of physiological distribution in some organs such as the brain and liver, and false negative results (i.e., after prolonged antibiotic therapy) limit the usefulness of ^18^F-FDG PET in this clinical setting [41].

Also, the presence of a cardiovascular implantable electronic device can be burdened by complications such as late infections, potentially associated with significant morbidity and mortality and requiring immediate treatment; some authors suggested that ^18^F-FDG PET/CT can be useful in the detection of infectious foci, due to its high sensitivity, easy repeatability and non-invasiveness [44].

### 2.6. Other Clinical Indications

For the reasons reported above, ^18^F-FDG PET/CT may have a more important role in managing osteomyelitis patients. This tracer is highly enhanced in chronic osteomyelitis cases because activated macrophages continue to accumulate ^18^F-FDG in chronic infection [45]. ^18^F-FDG PET has the highest diagnostic accuracy in chronic osteomyelitis, in comparison to bone scan, human-labeled leukocyte scan, and MRI [46].

Diabetic foot infection is among the most common complications of diabetes, frequently leading to serious sequelae such as amputation. The differentiation of osteomyelitis in diabetic foot from neuropathic osteoarthropathy is of the utmost importance; in fact, neuropathic osteoarthropathy demonstrates lower ^18^F-FDG metabolism compared to osteomyelitis. ^18^F-FDG PET/CT can show high sensitivity and specificity in the diagnosis of diabetic foot infection and the detection of associated osteomyelitis, while scintigraphy with labeled white blood cells still plays the main diagnostic role in this field [47].

### 2.7. Limits of ^18^F-FDG PET/CT in Infectious Diseases

Activated human leukocytes in an inflammatory disease are characterized by high glucose consumption and, consequently, highly enhance ^18^F-FDG in PET imaging.

The use of ^18^F-FDG PET/CT to diagnose a wide range of patients with infectious or inflammatory diseases is growing. Nevertheless, using ^18^F-FDG PET/CT in patients with such diseases presents some limits and pitfalls. We must consider that technical aspects linked to the acquisition protocol and quality imaging may underestimate the in vivo assessment of the disease under study. The power resolution limit of PET imaging is one of the most unfavorable aspects that needs to be taken into account, as it does not allow the identification of small infectious foci, as in the case of small vegetations at the root of acute infective endocarditis [41]. On the other hand, some organs, such as the liver and brain, present a high rate of physiological tracer distribution, which can hinder the recognition or the exact identification of a liver abscess [48] or encephalitis [49].

Moreover, all inflammatory and infectious diseases are linked to a more diffuse and less pathognomonic pattern of FDG uptake than malignant diseases [50]. However, to the best of our knowledge, patients referred to PET/CT with suspected infection or inflammation are rarely treatment-naïve and may have received varying doses of antibiotics, corticosteroids, or other immune-modulating drugs at the time of the scan.

All cited features lead to a higher rate of false positive ^18^F-FDG findings and also, in some cases, a lower sensitivity for the detection of active disease; we also must consider that other immune cells of the chronic inflammation, such as fibroblasts, may uptake ^18^F-FDG, thus potentially mimicking an infectious process. One of the major factors limiting the use of ^18^F-FDG PET in this field is the possibility of uptake in post-surgical procedures, occurring four weeks after a surgical intervention and/or acute injuries [51,52].

Finally, in some specific clinical indications such as the diagnosis of the diabetic foot infection, glucose serum levels at the time of the scan may negatively affect the sensitivity of the scan, causing false negative results.

In diabetic patients as well as in other subjects, the intrinsic properties of ^18^F-FDG as the analogue of glucose represent the major features in terms of diagnostic accuracy and, at the same time, the most relevant limit. In the last decade, the attention of researchers was focused on the development of novel molecular probes for the identification of infectious diseases. These novel molecular probes may be grouped by their molecular structures or their biological behavior. Due to the complexity of their mechanisms of action and their heterogeneity, we chose to describe the most important of these tracers, already tested for human use, with a classification based on the labeled nuclide rather than the involved biological molecule, to synthesize and classify all radiopharmaceuticals encountered in the literature.

## 3. ^11^C-Labeled Radiopharmaceuticals

**Figure 2:**
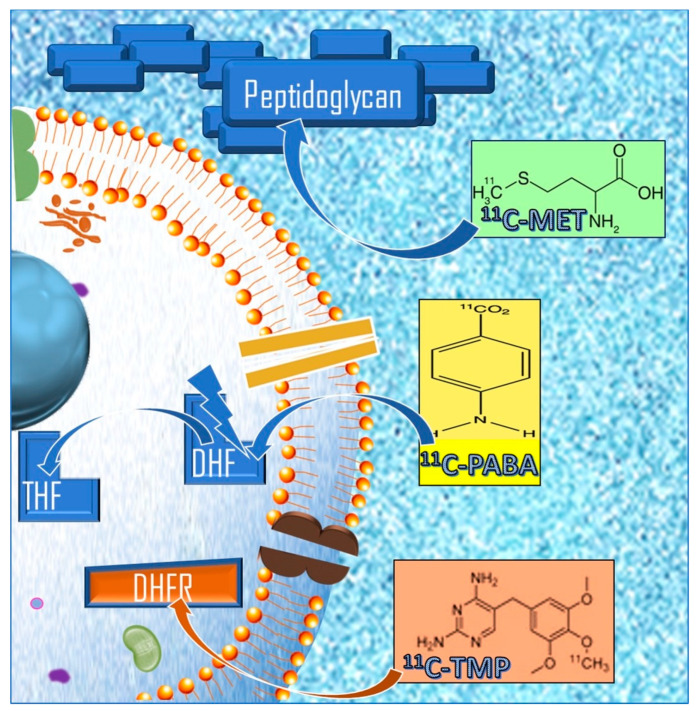
^11^C radiolabeled PET radiopharmaceuticals and their respective cellular targets in Gram-positive bacteria. DHFR: dihydrofolate reductase; DHF: dihydrofolate; THF: tetrahydrofolate; ^11^C-PABA: ^11^C-para-Aminobenzoic acid; D-^11^C-Met: D-methyl-^11^C-methionine; ^11^C-TMP: ^11^C-trimethoprim.

Carbon-11 (^11^C) is an artificial radioisotope of carbon and one of the most widely used radionuclides in the PET imaging pharmacopeia, as carbon is present in any organic molecule. The incorporation of carbon-11 does not change the properties of the recipient molecule. Furthermore, given the short half-life (20 min), it is possible to administer more tracers in vivo on the same day [53]. This chapter will discuss the most accredited ^11^C-radiotracers in PET molecular imaging of bacterial infections (Figure 2).

### 3.1. D-methyl-^11^C-methionine (D-^11^C-Met)

In an attempt to select biomolecules capable of selectively identifying bacterial infections, some small molecules metabolized exclusively by bacteria were synthesized as PET radiopharmaceuticals. In this direction, unnatural D-amino acids have shown to be very promising as they are incorporated into bacterial cell membranes, as opposed to their L-amino acid counterparts that are building blocks of proteins in most mammalian cells [54,55]. Peptidoglycan is a polymer assembled in one or more layers in the bacterial wall and gives the cell shape and size by fixing its components [56]. In vitro studies with fluorescent D-amino acids demonstrated highly specific, rapid incorporation into the bacterial wall (~30 s in *E. coli*) [57].

In the first clinical study applied to humans, bio-distribution, dosimetry, and diagnostic efficacy in infected orthopedic prostheses were evaluated using D-methyl-^11^C-methionine (D-^11^C-Met) on PET/MRI scans. After administration, the first organs in which activity was manifested were the heart, lungs, and kidneys (within 4 min) and then decreased (within 20 min); rapid renal clearance was observed, with slow hepatobiliary clearance. Of note was the low absorption in the gastrointestinal system, where the normal bacterial flora resides, and in the musculoskeletal system, with evidently a good signal-to-noise ratio at the target sites. Low effective doses were reported, lower than those of L-^11^C-Met, which is widely used in oncology imaging. D-^11^C-Met uptake was shown to be approximately 1.5-fold higher in prosthetic joints with suspected infection than in the contralateral non-prosthetic joints [58].

### 3.2. ^11^C-para-aminobenzoic Acid (^11^C-PABA)

The demonstration that ^11^C-para-aminobenzoic acid (^11^C-PABA) is incorporated into tetrahydrofolate via the bacterial folate pathway has created the conditions for the synthesis of radiolabeled molecules that can be used in nuclear medical imaging [59,60,61]. ^11^C-PABA is used as a substrate by dihydropteroate synthase in the folate synthesis pathway and is subsequently trapped within bacteria [59]. ^11^C-PABA rapidly accumulates in a broad range of pathogenic bacteria and their clinical strains, including multidrug-resistant organisms (MDRO), and is able to detect sites of infection in both the mouse model of *Escherichia coli* myositis and detect and identify *Staphylococcus aureus*. In a study with healthy human participants [62], ^11^C-PABA was safe and well tolerated with a favorable dosimetric profile; it also has renal excretion kinetics that make it applicable in functional studies [63]. Its background signal in the brain, lungs, and muscles is low, allowing prediction of an advantageous signal-to-noise ratio for infections at these anatomical sites.

### 3.3. ^11^C-trimethoprim (^11^C-TMP)

^11^C-trimethoprim (^11^C-TMP) is a broad-spectrum antibiotic that belongs to an inhibitory class of dihydrofolate reductase (DHFR), which is an important enzyme in the de novo pathway of purine and thymidine synthesis, essential for the survival of the microorganism [64]. ^11^C-labeled trimethoprim (TMP) antibiotic-derived PET radiotracers accumulate specifically at sites of bacterial infection, as opposed to sites of sterile inflammation or neoplasms [65,66]. Several examples of first-in-human ^11^C-TMP imaging cases are reported in the literature [67]. Regarding the antibiotic resistance issue, it was seen to not affect imaging because drug-resistant bacterial species had a similar uptake of ^11^C-TMP compared to non-resistant species [68].

## 4. ^68^Ga-Labeled Radiopharmaceuticals

Gallium-68 (^68^Ga)-labeled radiopharmaceuticals can be suitable for inflammation and infection imaging, considering their availability from a generator and ease of labeling. In the past decade, the development of ^68^Ga-labeled PET tracers has carved out an important place in the diagnostic panorama of inflammation and infection imaging.

In particular, PET imaging with these tracers offers the opportunity to follow the molecular and cellular processes of inflammation in vivo, such as receptor expression, chemotaxis, and macrophage activation.

Some of these radiopharmaceuticals are represented by ^68^Ga-citrate, ^68^Ga-Fibroblast Activation Protein Inhibitor (^68^Ga-FAPI), and ^68^Ga-labeled somatostatin analogues (^68^Ga-DOTATOC, ^68^Ga-DOTANOC, and ^68^Ga-DOTATATE).

### 4.1. ^68^Ga-Citrate

Being analogues of iron, the isotopes of Gallium accumulate at sites of infection and inflammation following several molecular mechanisms; ^67^Ga-citrate and ^68^Ga-citrate can be used for inflammation imaging with SPECT and PET, respectively.

Nevertheless, ^67^Ga-citrate scintigraphy and/or SPECT present some limits, such as long half-life decay (78 h), low image quality and resolution, and long duration of the diagnostic procedures (3 days) [69]. ^68^Ga is a useful beta-emitting isotope for PET imaging, produced by commercially available Germanium/Gallium generators, with a shorter half-life decay (67 min) [70].

**Figure 3:**
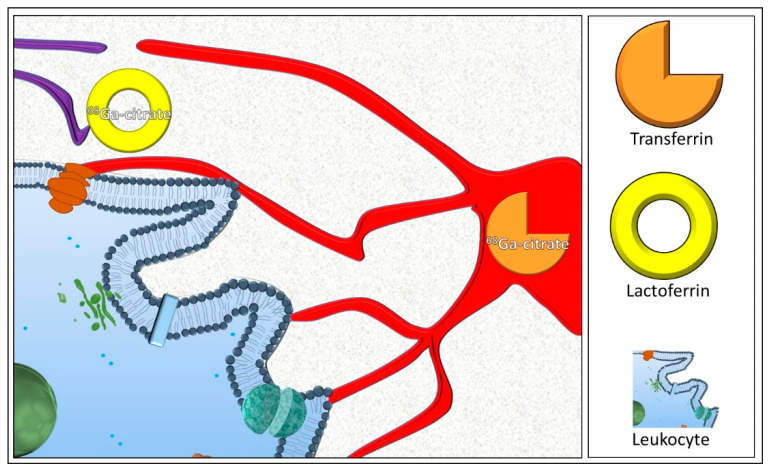
After intravenous administration, transferrin-bounded ^68^Ga-citrate circulates in the bloodstream and is transported by the arteries to the site of inflammation by mechanisms of blood flow and increased vascular permeability. The ferric ion-like properties of ^68^Ga-citrate allow its binding to lactoferrin, released by the apoptotic processes of leukocytes.

After the intravenous injection, ^68^Ga-citrate circulates in the bloodstream bounded with transferrin and is carried to sites of inflammation and infection by zonal blood flow and increased vascular permeability. Being a ferric ion-like nuclide, ^68^Ga-citrate binds to lactoferrin, released by the apoptotic processes of leukocytes (Figure 3).

The blood pool, liver, spleen, kidneys and growth plates were the most common sites of ^68^Ga-citrate involvement [71]. Thus, ^68^Ga-citrate is useful for imaging tuberculosis [72]; other feasible applications could be represented by bacterial infections [69,73], periprosthetic joint infections [74], and rheumatoid arthritis [75]. Further studies are needed to ensure its role in these clinical settings.

On a theoretical basis, ^68^Ga-citrate could be an alternative to ^18^F-FDG in infection imaging due to the peculiar characteristics of the nuclide in this clinical setting, already known for SPECT imaging, with the added value of a higher resolution limit provided by PET and better anatomical evaluation supplied by the co-registered CT component of the exam. However, cited studies are limited to a few researchers worldwide, while a direct comparison with ^18^F-FDG PET/CT concerning the diagnostic accuracy in large populations is still missing.

### 4.2. ^68^Ga-Fibroblast Activation Protein Inhibitor (^68^Ga-FAPI)

**Figure 4:**
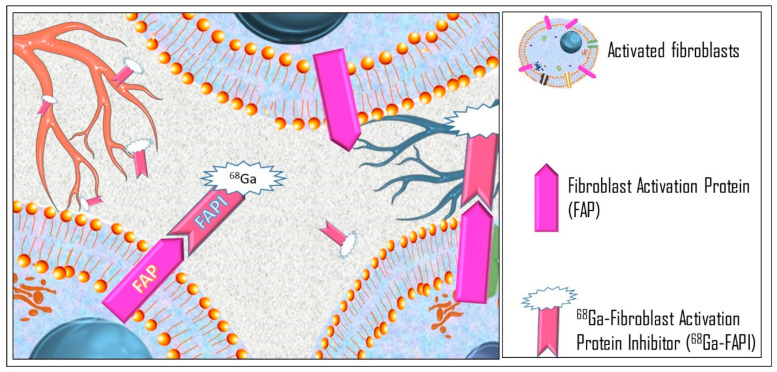
^68^Ga-FAPI reaches the infectious site by the bloodstream and interacts with FAP in fibroblasts of the infectious microenvironment.

The fibroblast activation protein (FAP) is a cell membrane protein overexpressed in cancer-associated fibroblasts, representing a component of the tumor stroma. Several clinical studies have highlighted the usefulness of gallium-labeled FAP inhibitor in imaging cancer cells due to the high-affinity complex with FAP (Figure 4), demonstrating ^68^Ga-FAPI PET/CT to be highly feasible [76,77], particularly because of the low physiological uptake in the human body, with high *target-to-background* ratio [78].

From the experience acquired with oncologic patients, the possibility has emerged to detect high ^68^Ga-FAPI uptake in sites not linked to the underlying disease [79]. Particularly, it has been shown that ^68^Ga-FAPI can be enhanced in inflammation and infections in cancer patients. A recent review of 108 papers evaluating FAPI labeled with either ^18^F and ^68^Ga showed that a large number of benign clinical entities may show FAPI uptake and should be considered when interpreting FAPI PET/CT in cancer patients, since FAPI uptake may be detected in arterial plaques, post-traumatic bone/articular lesions, arthritis, and in inflammation, infection, fibrosis, inflammatory/reactive lymph nodes and tuberculosis [80]. The authors hypothesized that these diagnostic pitfalls may occur from both specific uptake through rising FAP cellular expression and unspecific accumulation due to edema and inflammation processes. Moreover, it is also known that some cellular types such as activated fibroblasts show increased tracer accumulation, as in systemic sarcoidosis [81] or intra-cranial bacterial lesions [82]. However, the role of this tracer in infectious diseases is still unclear.

### 4.3. ^68^Ga-DOTATOC, ^68^Ga-DOTANOC, and ^68^Ga-DOTATATE

**Figure 5:**
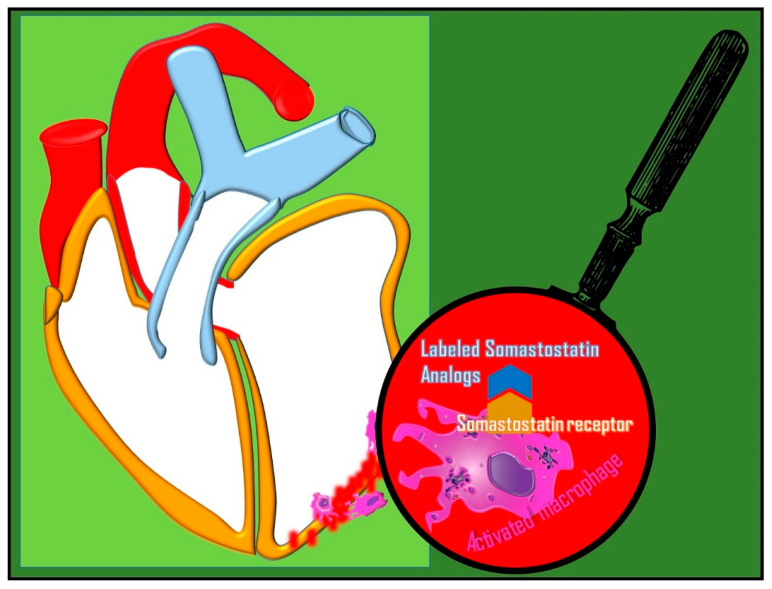
The uptake of radiolabeled somatostatin analogues in infection is due to the overexpression of cell surface somatostatin receptors in activated macrophages.

Some cellular types of inflammation such as activated macrophages and T cells express somatostatin receptors on the cell surface (Figure 5). Thus, radiolabeled somatostatin analogues may be enhanced at the sites of inflammation.

One of the main proposed applications of these tracers is linked to their capability to detect the phlogosis of atherosclerotic plaques, in both coronary and carotid arteries [83,84], with a higher signal-to-background ratio, in comparison with ^18^F-FDG due to the physiological myocardial uptake of glucose [41].

Another disease characterized by mild-to-high somatostatin analogue uptake is sarcoidosis, since somatostatin receptors are also expressed in sarcoid granulomas; some authors have proposed a feasible role for ^68^Ga-DOTATATE in the management of patients with active cardiac sarcoidosis despite a low signal-to-background ratio and underestimation of treatment response [85].

It has also emerged in case reports or preliminary focused studies in the literature that the uptake of radiolabeled somatostatin receptor analogues in inflammation is a condition that is not rare and may occur in patients with neuroendocrine tumors [86].

In particular, ^68^Ga-DOTANOC and ^68^Ga-DOTATOC uptake has been documented in acute myocarditis [87,88], and ^68^Ga-DOTATATE is enhanced in large vessel vasculitis, such as Takayasu arteritis [89].

However, due to the heterogeneous nature of these studies, it is difficult to pool the for statistical analysis. Data in the literature seem to support more versatile and better validated ^68^Ga-based inflammation probes, such as ^68^Ga-citrate and ^68^Ga-FAPI, mainly due to higher signal-to-background ratio and better diagnostic accuracy [90]. On this topic, it also has been shown that both ^18^F-FDG and ^68^Ga-DOTANOC uptake were similar in pulmonary tuberculosis; ^68^Ga-DOTANOC helped to detect pulmonary tuberculosis lesions, while ^18^F-FDG was more sensitive for both active and sub-clinical lesions [91].

In neuroendocrine tumor patients, incidental uptake of radiolabeled somatostatin receptor analogues linked to inflammation should be taken into account. In particular, cardiac uptake can indicate myocardial inflammation. Myocardial inflammation is a condition of clinical importance, and its underlying etiology should be further assessed to prompt eventual and necessary treatment, as it is linked to a poor prognosis [86].

## 5. Other ^18^F-Labeled Radiopharmaceuticals

Due to its physicochemical properties, ^18^F is a nuclide showing several advantages in PET imaging that are ideal for PET studies. The half-life of ^18^F (110 min), the most predominant radionuclide used in PET imaging, permits multi-step radiosynthetic protocols and tracer transport between clinical facilities. Moreover, the high positron (>97% β^+^) branching and low positron energy (0.635 MeV) of ^18^F provide high-resolution PET images [92].

### 5.1. ^18^F-FDG-Labeled White Blood Cells (^18^F-FDG-WBCs)

**Figure 6:**
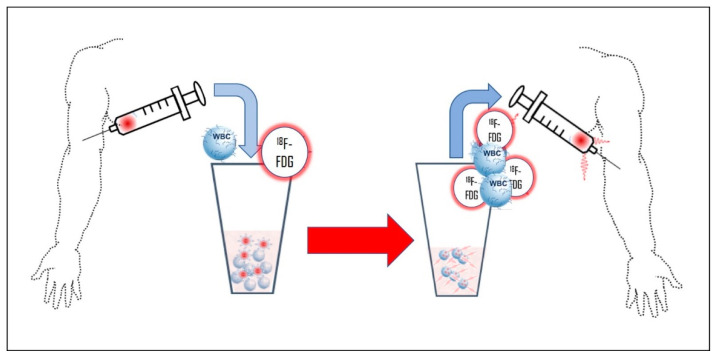
Simplified preparation of ^18^F-FDG-radiolabeled white blood cells. Venous blood is collected in a heparinized syringe; after plasma separation and centrifugation, ^18^F-FDG is added to the white blood cell pellet suspension. After incubation, the radiolabeled white blood cell sediment is reconstituted with the patient’s cell-free plasma and intravenously reinjected (^18^F-FDG: ^18^F-fluorodeoxyglucose;—WBC: white blood cell).

White blood cells (WBCs) can be labeled with ^18^F-FDG just as with gamma-emitting radiotracers (^111^In or ^99^mTc), with the added value of a co-registered, whole-body PET/CT scan, allowing a comprehensive evaluation of the entire body in a single session. The labeling methodology [93] involves the removal of red blood cells by sedimentation of heparinized peripheral blood and subsequent centrifugation of the WBC-enriched plasma to separate the WBC pellet. At this time, the labeling takes place by incubation with ^18^F-FDG (Figure 6).

**Figure 7:**
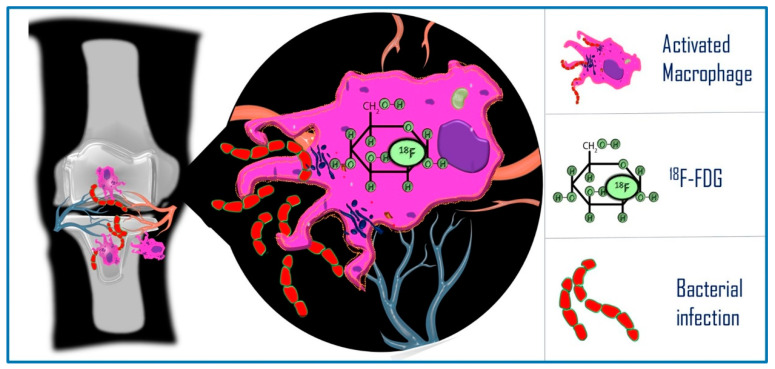
Mechanism of uptake of ^18^F-FDG-labeled white blood cells in infectious foci.

After administration, the optimal imaging time is 120 min post-injection (Figure 7).

Clinical applications of ^18^F-FDG-WBC [94] include infected renal cysts, infected peri-pancreatic collections, cardiac implant infections, or after cardiac surgery. In a pilot study, a feasible role was hypothesized in the management of chronic prosthetic joint infections, without meaningful differences with ^99m^Tc-HMPAO-WBC SPECT/CT in terms of diagnostic accuracy [21].

Another potential indication is the evaluation of bone involvement in diabetic cellulitis [95]. In prosthetic implant surgery, the use of ^18^F-FDG-WBC is not affected by the limits presented by triphasic bone scintigraphy, generally related to false positive findings from postoperative reactive changes [96]. Even though it is not a specific infection tracer, a negative ^18^F-FDG PET still has high negative predictive value; hence, in clinical practice, it is not necessary to further study leukocytes labeled with ^18^F-FDG [97].

Many diagnostic limitations are due to potential false negatives, such as chronic infections mediated by lymphocytes and macrophages or previous therapy (antibiotics, immune suppressants), reducing the immune response mediated by neutrophils; furthermore, there is low performance in the diagnosis of vertebral infections. From data available in literature reviews, the use of ^18^F-FDG-WBC PET/CT in infections demonstrates feasibility and good diagnostic performance but remains confined to the research domain, considering the difficulty in labeling and limited availability of data [94,98]; moreover, WBCs take 24 h to accumulate in infections, and the half-life of ^18^F is only 110 min [6].

### 5.2. ^18^F-FAPI

Numerous studies have demonstrated a superior diagnostic accuracy of FAPI PET/CT over FDG PET/CT in several malignant diseases. As already stated for ^68^Ga-FAPI, the cancer specificity of the ^8^F-labeled analogue remains understudied, with several cases of false-positive FAPI PET/CT reported in oncologic studies [80]. Due to its molecular affinity with ^68^Ga-FAPI, the possibility of ^18^F-FAPI uptake is also conceivable in further inflammatory conditions, other than arterial plaques and arteritis. Nevertheless, current data on this topic are limited to diagnostic pitfalls registered in cancer populations. ^18^F-FAPI needs to be tested for inflammation and infectious diseases.

### 5.3. ^18^F-fluoro-maltose (^18^F-FDM)

^18^F-fluoro-maltose (^18^F-FDM) can be obtained by the reaction of ^18^F-FDG with maltose phosphorylase; this tracer has showed in vivo accumulation directly into several clinically relevant bacteria. In particular, the high sensitivity of ^18^F-FDM methicillin-resistant Staphylococcus aureus could justify its clinical use in infected patients [99].

Thus, as already stated for D-^11^C-Met and ^11^C-PABA and ^11^C-TMP, ^18^F-FDM has the potential to directly image bacterial infections, being metabolized in bacteria. However, the role of this tracer in inflammation imaging needs to be investigated through in vivo studies [100].

### 5.4. ^18^F-fluorodeoxysorbitol (^18^F-FDS)

^18^F-Fluorodeoxysorbitol (^18^F-FDS) has already been tested in preclinical studies as an imaging probe with the capability to be enhanced in bacteria [101] and fungal diseases [102]; in healthy human volunteers, this tracer has shown no adverse reactions and the ability to specifically detect Enterobacteriaceae infections, showing suitability for human use from a radiation dosimetry perspective [103]. Unfortunately, no progress has been made in translating these results to routine human use.

### 5.5. ^18^F-fluoropropyl-trimethoprim (^18^F-FPTMP)

^18^F-fluoropropyl-trimethoprim (^18^F-FPTMP) is the fluorinated analogue of the above-mentioned ^11^C-TMP. As bacteria-specific imaging probes, both of these tracers are under study for their affinity for key enzymes in the folate synthesis pathway, in preclinical models and in humans [62]. Despite the promising role of all antibiotic tracers, limited success has been registered for ^18^F-FPTMP during the last decade, mainly due to limitations of PET spatial resolution in the detection of bacterial infections [104].

## 6. Discussion

The PET tracer most used in clinical practice for infectious disease is ^18^F-FDG, showing uptake in both infected and sterile inflammation, being enhanced in activated monocytes and macrophages. Among its advantages, we must consider the extreme versatility of this glucose analogue in imaging inflammation, the capability to assess the response to therapy, the easy reproducibility of related PET scans, its wide availability, and the extensive literature. The limits of ^18^F-FDG are its poor specificity, especially in detecting aseptic inflammation, requiring further diagnostic procedures, with effects on dosimetry [22].

The efforts of radiochemists are dedicated to overcoming these limits by developing novel radiopharmaceuticals such as those cited above (Table 1).

As bacterial imaging probes, proposed ^11^C-labeled tracers can present a higher specificity intrinsically linked to the molecular pathways of D-^11^C-Met, ^11^C-PABA, and ^11^C-TMP. They could improve the detection of infectious foci, allowing the visualization of bacteria directly at sites of infection [104].

Beyond good theoretical specificity, the major limit of these bacterial tracers could be the difficulty to be enhanced in a large volume of bacteria, due to reports of engulfed bacteria inside macrophages, in vitro testing results [105], and also the suboptimal limits of PET power resolution, corresponding to 5–10 mm [106]; moreover, the concentration of this kind of tracer in the bacteria should be higher than that of the surrounding background to overcome these limits.

On the other hand, thanks to their peculiar molecular pathways, the possible role of these radiopharmaceuticals in ensuring the resistance of certain bacteria to antimicrobial drugs needs to be assessed. Antimicrobial resistance is still a diagnostic dilemma, in terms of best treatment options and patient prognosis.

In this specific clinical setting, the role of PET with bacterial tracers needs to be investigated in order to avoid unnecessary antibiotic treatments and to choose the best antimicrobial drug [107]. The uptake of these tracers in bacterial infections, detected by PET, could be a preliminary step prior to submitting patients to a specific antimicrobial drug [108].

All of the above considerations can be applied also to the fluorinated counterparts of antibiotic tracers, respectively: ^18^F-FPTMP, ^18^F-FDS and ^18^F-FDM. The longer half-life decay of ^18^F could enable the availability of these tracers also in those PET centers not provided by a cyclotron, also allowing both early and late imaging to improve research results [109]. Basically, there is still a lack of knowledge about the potentiality of this class of PET radiopharmaceuticals.

Conversely, data in the literature about some of the ^68^Ga-labeled tracers for inflammation and infection are more robust. In particular, ^68^Ga-Citrate has already shown the capability to detect bacterial infections [72,74] due to the possibility of being highly enhanced in infectious foci and surrounding tissues, since its binding to transferrin allows the distribution in the infected tissue, which is characterized by high vascular permeability and chemotaxis factors produced by human leukocytes [69,71].

^68^Ga-labeled somatostatin analogues and ^68^Ga-FAPI enable visualization of the inflammatory process and infectious foci, although the record of these findings is normally linked to incidental observations during whole-body PET/CT scans performed for oncologic purposes [79], particularly in neuroendocrine tumor patients [86].

All ^68^Ga-labeled tracers share similar metabolic conditions such as macrophage activation, receptor expression, and chemotaxis, allowing their feasible use in the management of infection. ^18^F-FAPI permits tracking other cell types such as fibroblasts, potentially improving specificity, but this feature has only been incidentally documented in oncologic studies. Surely, ^68^Ga-labeled tracers show high *target-to-background* ratios and share a low rate of physiological distribution in the brain, myocardium and liver, thus potentially playing a role in overcoming limitations of ^18^F-FDG in brain infections, myocarditis and liver abscess. ^68^Ga-DOTATOC and DOTANOC have already shown good diagnostic performance in diagnosing acute myocarditis [87,88], while ^68^Ga-FAPI may enable the identification of intracranial infections [82]. A further feasible application of ^68^Ga-labeled somatostatin analogues may also be represented by the imaging of active atherosclerotic plaques.

It is also necessary not to underestimate the larger experience of the nuclear medicine community with ^68^Ga-citrate, due to its counterpart labeled with ^67^Ga, which was the most important tracer for the study of infection and phlogosis by SPECT before the advent of the PET era. On this topic, we must consider the potential impact of ^68^Ga-citrate imaging of infectious diseases with the help of the better-performing PET scanner, provided with correlative CT for better delineation and stratification of infectious foci, in a single whole-body imaging session.

Concerning the other fluorinated PET tracers, we must clarify that few data support a high specificity of ^18^F-FDG-WBC in the diagnosis of infectious foci. Unfortunately, this is a time-consuming approach, also requiring the handling of blood, with limited data in the literature. ^18^F-FAPI can present features similar to those of ^68^Ga-FAPI with a better-quality image due to the ^18^F emission [110]. Unfortunately, the data available in the literature do not allow a comprehensive evaluation.

Despite the heterogeneity of the proposed tracers, we can suggest some considerations.

To date, ^18^F-FDG remains the most versatile and useful tracer for the diagnosis of infection. On the other hand, ^68^Ga-labeled tracers could overcome the limits of the specificity of ^18^F-FDG, also enabling visualization of infectious foci in the brain and myocardium. The ^68^Ga-labeled somatostatin analogues can represent a valid alternative for the diagnosis of myocarditis.

The antibiotic tracers, especially those labeled with ^18^F, can play a role in the management of bacterial infections with possible implications for the assessment of treatment efficacy, in particular in the field of antidrug resistance in bacteria; nevertheless, the limited experience and limitations due to the low power resolution of PET do not support their use in humans. Among nuclides, ^18^F is the more favorable in terms of quality image and availability [110,111]; ^11^C presents good-quality images but a rapid half-life decay, limiting its wide distribution, while ^68^Ga-labeled tracers present an advantage over ^18^F-FDG in that they can be created by a ^68^Ge/^68^Ga generator, thus not requiring a nearby cyclotron for their use [112]. As a future trend, Zirconium-89 (^89^Zr) is worthy of note as an emerging radioisotope produced by cyclotrons, with a long half-life (3.3 days) suitable for immuno-PET (with the synthesis of ^89^Zr-antibodies) [113]. Its potential application is represented by the production of radiolabeled antibodies directed against bacterial surface molecules. An example of these attractive radiopharmaceuticals is the radiolabeled human monoclonal antibody ^89^Zr-SAC55, directed against lipoteichoic acid (LTA), a molecule localized on the surface of Gram-positive bacteria such as *Staphylococcus aureus*, as demonstrated by Pickett et al. in a preclinical study; ^89^Zr-SAC55 may be useful in the identification of infection sites, helping in the discrimination between sterile inflammation and infection [114].

However, in the literature, there is still a lack of head-to-head comparison studies among ^18^F-FDG and other cited tracers. Few data are available for all radiopharmaceuticals evaluated in this clinical setting, in comparison to the robust literature developed in the last decade on ^18^F-FDG PET/CT in infectious diseases.

As a future trend, we must also consider that some of the above limits can be overcome by the advent of PET/magnetic resonance imaging (MRI) in clinical practice. PET/MRI, with higher soft-tissue contrast and better-performing PET scanners, will extend the field of molecular imaging to the study of infectious diseases [115], with high sensitivity [116]. This is a necessary step to perform prior to considering, as a further point of reflection, that further imaging probes are under study for the visualization of retroviral infections [117], while ^18^F-FDG already plays a role in the detection of tracer-avid lymphadenopathies linked to monkeypox infection in HIV patients [118].

## 7. Conclusions

^18^F-FDG is still the best tracer in the imaging of infections, but some novel molecular probes can overcome its limits.

^68^Ga-labeled PET radiopharmaceuticals can play a role in the diagnosis of infectious diseases due to their uptake mechanisms and the high *target-to-background* ratio expressed. Among these, ^68^Ga-citrate shows encouraging results in the detection of infections, while ^68^Ga-labeled somatostatin analogues can help in diagnosing myocarditis and other infectious foci in tissues with a high physiological gradient of ^18^F-FDG uptake. ^18^F could be the best nuclide for imaging; among fluorinated tracers, while antibiotic radiopharmaceuticals need to be further investigated. Similar findings are conceivable for ^11^C-labeled antibiotic tracers, despite the less favorable availability of ^11^C.

Head-to-head comparison studies are of the utmost importance to determine the best alternative to ^18^F-FDG.

## Figures and Tables

**Table 1:** Summary of the different radiopharmaceuticals used for infectious disease imaging, with their respective molecular pathways, clinical settings, advantages and drawbacks.

*Nuclide*	*Radiopharmaceuticals*	*Molecular Pathway*	*Evaluated Disease*	*Advantages*	*Drawbacks*
	D-^11^C-Met	Incorporation into bacterial cell membrane	Infected joint prostheses [58]	Uptake in bacteria, *high signal-to-background* ratio	Limits of PET spatial resolution for the detection of bacteria
*^11^* *C*	^11^C-PABA	Bacterial folate synthesis	Methicillin-resistant S. aureus infection [62]	Uptake in bacteria, *high signal-to-background* ratio	Limits of PET spatial resolution for the detection of bacteria
	^11^C-TMP	Bacterial purine and thymidine synthesis	Bacterial infection [67,68]	Uptake in bacteria	Limits of PET spatial resolution for the detection of bacteria
					
	^68^Ga-Citrate	Leukocytes apoptosis	Tuberculosis [72], bacterial infections [69,73], periprosthetic joint infections [74], and rheumatoid arthritis [75]	High affinity for infection sites and *signal-to-background* ratio	Missing direct comparison with ^18^F-FDG
*^68^* *Ga*	^68^Ga-FAPI	FAP expression in fibroblast	Sarcoidosis [81], intracranial bacterial lesions [82]	Selectivity for fibroblasts, high *signal-to-background* ratio	Few available data
	^68^Ga-DOTATOC/DOTANOC/DOTATATE	Somatostatin receptor expression in macrophages and T-cells	Atherosclerotic plaques [83,84], cardiac sarcoidosis [85], myocarditis [87,88], vasculitis [89]	Somatostatin receptors exression in granulomas	Mostly incidental detection in PET/CT scans developed for oncologic purpose
					
	^18^F-FDG-WBC	Imaging of white blood cells	Infected renal cysts, infected peri-pancreatic collections, cardiac implant infections [94]	Direct visualization of leukocytes in infected foci	False positive findings, difficulty in labeling
	^18^F-FAPI	FAP expression in fibroblast	Arterial plaques and arteritis [80]	Selectivity for fibroblasts	Incidental detection in PET/CT developed for oncologic purpose
*^18^* *F*	^18^F-FDM	Accumulation into bacteria	Methicillin-resistant S.aureus infection [99]	High sensitivity	Few available data
	^18^F-FDS	Accumulation into bacteria and fungi	Bacterial diseases and mycoses [102]	Detection of Enterobacteriaceae	Only preclinical studies
	18F-FPTMP	Bacterial folate synthesis	S.aureus infection [62]	High affinity for bacteria	Limits of PET spatial resolution for the detection of bacteria

## Data Availability

No new data were created or analyzed in this study. Data sharing is not applicable to this article.

## References

[1] Rudd K.E., Johnson S.C., Agesa K.M., Shackelford K.A., Tsoi D., Kievlan D.R., Colombara D.V., Ikuta K.S., Kisoon N., Finfer S. (2020). Global, regional, and national sepsis incidence and mortality, 1990–2017: Analysis for the Global Burden of Disease Study. Lancet.

[2] Gotthardt M., Bleeker-Rovers C.P., Boerman O.C., Oyen W.J.G. (2013). Imaging of inflammation by PET, conventional scintigraphy, and other imaging techniques. J. Nucl. Med. Technol..

[3] Filippi L., Uccioli L., Giurato L., Schillaci O. (2009). Diabetic foot infection: Usefulness of SPECT/CT for 99mTc-HMPAO-labeled leukocyte imaging. J. Nucl. Med..

[4] Yoo J., Cheon M. (2022). Septic Pulmonary Emboli Detected by 18F-FDG PET/CT in a Patient with Central Venous Catheter-Related Staphylococcus aureus Bacteremia. Diagnostics.

[5] Hess S. (2023). [18F]FDG-PET/CT in patients with bacteremia: Clinical impact on patient management and outcome. Front. Med..

[6] Lauri C., Signore A., Glaudemans A.W.J.M., Treglia G., Gheysens O., Slart R.H.J.A., Iezzi R., Prakken N.H.J., Debus E.S., Honig S. (2022). Evidence-based guideline of the European Association of Nuclear Medicine (EANM) on imaging infection in vascular grafts. Eur. J. Nucl. Med. Mol. Imaging.

[7] Palestro C.J. (2023). Molecular Imaging of Periprosthetic Joint Infections. Semin. Nucl. Med..

[8] Ten Hove D., Wahadat A.R., Slart R.H.J.A., Wouthuyzen-Bakker M., Mecozzi G., Damman K., Witteveen H., Caliskan K., Manintveld O.C., Sinha B. (2023). Added value of semi-quantitative analysis of [18F]FDG PET/CT for the diagnosis of device-related infections in patients with a left ventricular assist device. Eur. Heart J. Cardiovasc. Imaging.

[9] Betrains A., Boeckxstaens L., Moreel L., Wright W.F., Blockmans D., Van Laere K., Vanderschueren S. (2023). Higher diagnostic yield of 18F-FDG PET in inflammation of unknown origin compared to fever of unknown origin. Eur. J. Intern. Med..

[10] Régis C., Benali K., Rouzet F. (2023). FDG PET/CT Imaging of Sarcoidosis. Semin. Nucl. Med..

[11] Ferro P., Boni R., Bartoli F., Lazzeri F., Slart R.H.J.A., Erba P.A. (2023). Radionuclide Imaging of Infective Endocarditis. Cardiol. Clin..

[12] Calabria F., Bagnato A., Guadagnino G., Toteda M., Lanzillotta A., Cardei S., Tavolaro R., Leporace M. (2023). COVID-19 vaccine related hypermetabolic lymph nodes on PET/CT: Implications of inflammatory findings in cancer imaging. Oncol. Res..

[13] Griffin M.T., Werner T.J., Alavi A., Revheim M.E. (2023). The value of FDG-PET/CT imaging in the assessment, monitoring, and management of COVID-19. Eur. Phys. J. Plus.

[14] Kung B.T., Seraj S.M., Zadeh M.Z., Rojulpote C., Kothekar E., Ayubcha C., Ng K.S., Ng K.K., Au-Yong T.K., Werner T.J. (2019). An update on the role of 18F-FDG-PET/CT in major infectious and inflammatory diseases. Am. J. Nucl. Med. Mol. Imaging.

[15] Jørgensen N.P., Alstrup A.K., Mortensen F.V., Knudsen K., Jakobsen S., Madsen L.B., Bender D., Breining P., Petersen M.S., Schleimann M.H. (2017). Cholinergic PET imaging in infections and inflammation using 11C-donepezil and 18F-FEOBV. Eur. J. Nucl. Med. Mol. Imaging.

[16] Suilamo S., Li X.G., Lankinen P., Oikonen V., Tolvanen T., Luoto P., Viitanen R., Saraste A., Seppänen M., Pirilä L. (2022). 68Ga-Citrate PET of Healthy Men: Whole-Body Biodistribution Kinetics and Radiation Dose Estimates. J. Nucl. Med..

[17] Kwee T.C., Kwee R.M., Alavi A. (2008). FDG-PET for diagnosing prosthetic joint infection: Systematic review and meta-analysis. Eur. J. Nucl. Med. Mol. Imaging.

[18] Rachh S.S., Basu S., Alavi A. (2022). Fluorodeoxyglucose PET/Computed Tomography in Evaluation of Prosthetic Joints and Diabetic Foot: A Comparative Perspective with Other Functional Imaging Modalities. PET Clin..

[19] Loharkar S., Basu S. (2023). PET-Computed Tomography in Bone and Joint Infections. PET Clin..

[20] Basu S., Kwee T.C., Saboury B., Garino J.P., Nelson C.L., Zhuang H., Parsons M., Chen W., Kumar R., Salavati A. (2014). FDG PET for diagnosing infection in hip and knee prostheses: Prospective study in 221 prostheses and subgroup comparison with combined (111)In-labeled leukocyte/(99m)Tc-sulfur colloid bone marrow imaging in 88 prostheses. Clin. Nucl. Med..

[21] Teiler J., Ahl M., Åkerlund B., Brismar H., Holstensson M., Gabrielson S., Hedlund H., Axelsson R. (2022). 99mTc-HMPAO-WBC SPECT/CT versus 18F-FDG-WBC PET/CT in chronic prosthetic joint infection: A pilot study. Nucl. Med. Commun..

[22] Khalid V., Schønheyder H.C., Larsen L.H., Nielsen P.T., Kappel A., Thomsen T.R., Aleksyniene R., Lorenzen J., Ørsted I., Simonsen O. (2020). Multidisciplinary Diagnostic Algorithm for Evaluation of Patients Presenting with a Prosthetic Problem in the Hip or Knee: A Prospective Study. Diagnostics.

[23] Divakaran S. (2023). Radionuclide Assessment of Sarcoidosis. Cardiol. Clin..

[24] Aide N., Benayoun M., Kerrou K., Khalil A., Cadranel J., Talbot J.N. (2007). Impact of [18F]-fluorodeoxyglucose ([18F]-FDG) imaging in sarcoidosis: Unsuspected neurosarcoidosis discovered by [18F]-FDG PET and early metabolic response to corticosteroid therapy. Br. J. Radiol..

[25] Chen H., Jin R., Wang Y., Li L., Li K., He Y. (2018). The Utility of 18F-FDG PET/CT for Monitoring Response and Predicting Prognosis after Glucocorticoids Therapy for Sarcoidosis. BioMed Res. Int..

[26] Goletti D., Pisapia R., Fusco F.M., Aiello A., Van Crevel R. (2023). Epidemiology, pathogenesis, clinical presentation and management of TB in patients with HIV and diabetes. Int. J. Tuberc. Lung Dis..

[27] Lawal I.O., Abubakar S., Ankrah A.O., Sathekge M.M. (2023). Molecular Imaging of Tuberculosis. Semin. Nucl..

[28] Kim D.W., Park S.A., Kim M.H. (2023). The utility of F-18 FDG PET/CT for diagnosis and response evaluation of hepatosplenic tuberculosis. Liver Int..

[29] Dhingra V.K., Khan D., Kumar R., Basu S. (2022). Nonmalignant Thoracic Disorders: An Appraisal of Fluorodeoxyglucose and Non-fluorodeoxyglucose PET/Computed Tomography Applications. PET Clin..

[30] Cimini A., Ricci M., Gigliotti P.E., Pugliese L., Chiaravalloti A., Danieli R., Schillaci O. (2021). Medical Imaging in the Diagnosis of Schistosomiasis: A Review. Pathogens.

[31] Danve A., O’Dell J. (2015). The Role of 18F Fluorodeoxyglucose Positron Emission Tomography Scanning in the Diagnosis and Management of Systemic Vasculitis. Int. J. Rheum. Dis..

[32] Webb M., Chambers A., AL-Nahhas A., Mason J.C., Maudlin L., Rahman L., Frank J. (2004). The role of 18F-FDG PET in characterising disease activity in Takayasu arteritis. Eur. J. Nucl. Med. Mol. Imaging.

[33] Narváez J., Estrada P., Vidal-Montal P., Sánchez-Rodríguez I., Sabaté-Llobera A., Nolla J.M., Cortés-Romera M. (2023). Impact of previous glucocorticoid therapy on diagnostic accuracy of [18F] FDG PET-CT in giant cell arteritis. Semin. Arthritis Rheum..

[34] Ramachandran A., Antala D., Pudasainee P., Panginikkod S. (2023). Positron Emission Tomography (PET) Scan as a Diagnostic Tool for Giant Cell Arteritis. Cureus.

[35] Chen J., Xu D., Sun W., Wang W.X., Xie N.N., Ruan Q.R., Song J.X. (2023). Differential diagnosis of lymphoma with 18F-FDG PET/CT in patients with fever of unknown origin accompanied by lymphadenopathy. J. Cancer Res. Clin. Oncol..

[36] Palestro C.J., Brandon D., Dibble E.H., Keidar Z., Kwak J. (2023). FDG PET in Evaluation of Patients With Fever of Unknown Origin: AJR Expert Panel Narrative Review. AJR Am. J. Roentgenol..

[37] Letertre S., Fesler P., Zerkowski L., Picot M.C., Ribstein J., Guilpain P., Le Moing V., Mariano-Goulart D., Roubille C. (2021). Place of the 18F-FDG-PET/CT in the Diagnostic Workup in Patients with Classical Fever of Unknown Origin (FUO). J. Nucl. Med..

[38] Tsai H.Y., Lee M.H., Wan C.H., Yang L.Y., Yen T.C., Tseng J.R. (2018). C-reactive protein levels can predict positive 18F-FDG PET/CT findings that lead to management changes in patients with bacteremia. J. Microbiol. Immunol. Infect..

[39] Khalatbari H., Shulkin B.L., Parisi M.T. (2023). Emerging Trends in Radionuclide Imaging of Infection and Inflammation in Pediatrics: Focus on FDG PET/CT and Immune Reactivity. Semin. Nucl. Med..

[40] Jain L., Mackenzie S., Bomanji J.B., Shortman R., Noursadeghi M., Edwards S.G., Miller R.F. (2018). 18F-Fluorodeoxyglucose positron emission tomography-computed tomography imaging in HIV-infected patients with lymphadenopathy, with or without fever and/or splenomegaly. Int. J. STD AIDS.

[41] Mikail N., Hyafil F. (2021). Nuclear Imaging in Infective Endocarditis. Pharmaceuticals.

[42] Wang T.K.M., Sánchez-Nadales A., Igbinomwanhia E., Cremer P., Griffin B., Xu B. (2020). Diagnosis of Infective Endocarditis by Subtype Using (18)F-Fluorodeoxyglucose Positron Emission Tomography/Computed Tomography: A Contemporary Meta-Analysis. Circ. Cardiovasc. Imaging.

[43] Gomes A., Glaudemans A., Touw D.J., van Melle J.P., Willems T.P., Maass A.H., Natour E., Prakken N.H.J., Borra R.J.H., van Geel P.P. (2017). Diagnostic value of imaging in infective endocarditis: A systematic review. Lancet Infect. Dis..

[44] Rubini G., Ferrari C., Carretta D., Santacroce L., Ruta R., Iuele F., Lavelli V., Merenda N., D’Agostino C., Sardaro A. (2020). Usefulness of 18F-FDG PET/CT in Patients with Cardiac Implantable Electronic Device Suspected of Late Infection. J. Clin. Med..

[45] Koort J.K., Mäkinen T.J., Knuuti J., Jalava J., Aro H.T. (2004). Comparative 18F-FDG PET of experimental Staphylococcus aureus osteomyelitis and normal bone healing. J. Nucl. Med..

[46] Basu S., Chryssikos T., Moghadam-Kia S., Zhuang H., Torigian D.A., Alavi A. (2009). Positron emission tomography as a diagnostic tool in infection: Present role and future possibilities. Semin. Nucl. Med..

[47] Gnanasegaran G., Vijayanathan S., Fogelman I. (2012). Diagnosis of infection in the diabetic foot using (18)F-FDG PET/CT: A sweet alternative?. Eur. J. Nucl. Med. Mol. Imaging.

[48] Fujimoto K., Norikane T., Mitamura K., Yamamoto Y., Okano K., Suzuki Y., Nishiyama Y. (2021). Liver Abscess With High 18F-FDG Uptake and No 18F-Fluorothymidine Uptake. Clin. Nucl. Med..

[49] Nabizadeh F., Ramezannezhad E., Sardaripour A., Seyedi S.A., Salehi N., Rezaeimanesh N., Naser Moghadasi A. (2022). [18F]FDG brain PET and clinical symptoms in different autoantibodies of autoimmune encephalitis: A systematic review. Neurol. Sci..

[50] Pijl J.P., Nienhuis P.H., Kwee T.C., Glaudemans A.W.J.M., Slart R.H.J.A., Gormsen L.C. (2021). Limitations and Pitfalls of FDG-PET/CT in Infection and Inflammation. Semin. Nucl. Med..

[51] Meyer M., Gast T., Raja S., Hubner K. (1994). Increased F-18 FDG accumulation in an acute fracture. Clin. Nucl. Med..

[52] Zhuang H., Sam J.W., Chacko T.K., Duarte P.S., Hickeson M., Feng Q., Nakhoda K.Z., Guan L., Reich P., Altimari S.M. (2003). Rapid normalization of osseous FDG uptake following traumatic or surgical fractures. Eur. J. Nucl. Med. Mol. Imaging.

[53] Goud N.S., Bhattacharya A., Joshi R.K., Nagaraj C., Bharath R.D., Kumar P. (2021). Carbon-11: Radiochemistry and Target-Based PET Molecular Imaging Applications in Oncology, Cardiology, and Neurology. J. Med. Chem..

[54] Neumann K.D., Villanueva-Meyer J.E., Mutch C.A., Flavell R.R., Blecha J.E., Kwak T., Sriram R., VanBrocklin H.F., Rosenberg O.S., Ohliger M.A. (2017). Imaging active infection in vivo using D-amino acid derived PET radiotracers. Sci. Rep..

[55] Mota F., Jain S.K. (2020). Flagging bacteria with radiolabeled D-amino acids. ACS Cent. Sci..

[56] Bugg T.D., Walsh C.T. (1992). Intracellular steps of bacterial cell wall peptidoglycan biosynthesis: Enzymology, antibiotics, and antibiotic resistance. Nat. Prod. Rep..

[57] Kuru E., Tekkam S., Hall E., Brun Y.V., Van Nieuwenhze M.S. (2015). Synthesis of fluorescent D-amino acids and their use for probing peptidoglycan synthesis and bacterial growth in situ. Nat. Protoc..

[58] Polvoy I., Seo Y., Parker M., Stewart M., Siddiqua K., Manacsa H.S., Ravanfar V., Blecha J., Hope T.A., Vanbrocklin H. (2022). Imaging joint infections using D-methyl-11C-methionine PET/MRI: Initial experience in humans. Eur. J. Nucl. Med. Mol. Imaging.

[59] Ordonez A.A., Weinstein E.A., Bambarger L.E., Saini V., Chang Y.S., DeMarco V.P., Klunk M.H., Urbanowski M.E., Moulton K.L., Murawski A.M. (2017). A Systematic Approach for Developing Bacteria-Specific Imaging Tracers. J. Nucl. Med..

[60] Mutch C.A., Ordonez A.A., Qin H., Parker M., Bambarger L.E., Villanueva-Meyer J.E., Blecha J., Carroll V., Taglang C., Flavell R. (2018). [11C]Para-Aminobenzoic Acid: A Positron Emission Tomography Tracer Targeting Bacteria-Specific Metabolism. ACS Infect. Dis..

[61] Zhang Z., Ordonez A.A., Wang H., Li Y., Gogarty K.R., Weinstein E.A., Daryaee F., Merino J., Yoon G.E., Kalinda A.S. (2018). Positron Emission Tomography Imaging with 2-[18F]F-p-Aminobenzoic Acid Detects Staphylococcus aureus Infections and Monitors Drug Response. ACS Infect. Dis..

[62] Ordonez A.A., Parker M.F., Miller R.J., Plyku D., Ruiz-Bedoya C.A., Tucker E.W., Luu J.M., Dikeman D.A., Lesniak W.G., Holt D.P. (2022). 11C-Para-aminobenzoic acid PET imaging of *S. aureus* and MRSA infection in preclinical models and humans. JCI Insight.

[63] Ruiz-Bedoya C.A., Ordonez A.A., Werner R.A., Plyku D., Klunk M.H., Leal J., Lesniak W.G., Holt D.P., Dannals R.F., Higuchi T. (2020). 11C-PABA as a PET Radiotracer for Functional Renal Imaging: Preclinical and First-in-Human Study11. J. Nucl. Med..

[64] Srinivasan B., Tonddast-Navaei S., Roy A., Zhou H., Skolnick J. (2019). Chemical space of Escherichia coli dihydrofolate reductase inhibitors: New approaches for discovering novel drugs for old bugs. Med. Res. Rev..

[65] Sellmyer M.A., Lee I., Hou C., Weng C.C., Li S., Lieberman B.P., Zeng C., Mankoff D.A., Mach R.H. (2017). Bacterial infection imaging with [18F]fluoropropyl-trimethoprim. Proc. Natl. Acad. Sci. USA.

[66] Sellmyer M.A., Lee I., Hou C., Lieberman B.P., Zeng C., Mankoff D.A., Mach R.H. (2017). Quantitative PET Reporter Gene Imaging with [11C]Trimethoprim. Mol. Ther..

[67] Gouws A.C., Kruger H.G., Gheysens O., Zeevaart J.R., Govender T., Naicker T., Ebenhan T. (2022). Antibiotic-Derived Radiotracers for Positron Emission Tomography: Nuclear or “Unclear” Infection Imaging?. Angew. Chem. Int. Ed. Engl..

[68] Lee I.K., Jacome D.A., Cho J.K., Tu V., Young A.J., Dominguez T., Northrup J.D., Etersque J.M., Lee H.S., Ruff A. (2022). Imaging Sensitive and Drug-Resistant Bacterial Infection with [11C]-TMP: In Vitro and First-in-Human Evaluation. J. Clin. Investig..

[69] Nanni C., Errani C., Boriani L., Fantini L., Ambrosini V., Boschi S., Rubello D., Pettinato C., Mercuri M., Gasbarrini A. (2010). 68Ga-citrate PET/CT for evaluating patients with infections of the bone: Preliminary results. J. Nucl. Med..

[70] Zimmerman B.E., Cessna J.T., Fitzgerald R. (2008). Standardization of (68)Ge/(68)Ga Using Three Liquid Scintillation Counting Based Methods. J. Res. Natl. Inst. Stand. Technol..

[71] Uğur A., Gültekin A. (2021). Physiological Animal Imaging with 68Ga-Citrate. Curr. Radiopharm..

[72] Vorster M., Maes A., Jacobs A., Malefahlo S., Pottel H., Van de Wiele C., Sathekge M.M. (2014). Evaluating the possible role of 68Ga-citrate PET/CT in the characterization of indeterminate lung lesions. Ann. Nucl. Med..

[73] Kumar V., Boddeti D.K., Evans S.G., Angelides S. (2012). (68)Ga-Citrate-PET for diagnostic imaging of infection in rats and for intra-abdominal infection in a patient. Curr. Radiopharm..

[74] Xu T., Zeng Y., Yang X., Liu G., Lv T., Yang H., Jiang F., Chen Y. (2022). Application of 68Ga-citrate PET/CT for differentiating periprosthetic joint infection from aseptic loosening after joint replacement surgery. Bone Jt. Res..

[75] Wang Z., Hou Y., Cai L., Chen Y. (2021). The Evaluation of 68Ga-Citrate PET/CT Imaging for Dihydroartemisinin in the Treatment of Rheumatoid Arthritis. Mol. Imaging Biol..

[76] Sayiner Z.A., Elboğa U., Sahin E., Ozturk S., Cayirli Y.B., Celen Y.Z., Akarsu E., Dogan I., Kilbas B., Eryilmaz K. (2023). Comparison of 68Ga-FAPI-04 and 18F-FDG PET/CT for diagnosis of metastatic lesions in patients with recurrent papillary thyroid carcinoma. Hell. J. Nucl. Med..

[77] Kessler L. (2023). Fibroblast Activation Protein Inhibitor (FAPI)-PET Imaging in Sarcoma. PET Clin..

[78] Wass G., Clifford K., Subramaniam R.M. (2023). Evaluation of the Diagnostic Accuracy of FAPI PET/CT in oncologic Studies: Systematic Review and Metaanalysis. J. Nucl. Med..

[79] Meetschen M., Sandach P., Darwiche K., Theegarten D., Moter A., Schaarschmidt B.M., Herrmann K., Fendler W.P., Hautzel H., Opitz M. (2023). Rabbit fever: Granulomatous inflammation by Francisella tularensis mimics lung cancer in dual tracer 18FDG and 68Ga-FAPI PET/CT. Eur. J. Nucl. Med. Mol. Imaging.

[80] Bentestuen M., Al-Obaydi N., Zacho H.D. (2023). FAPI-avid nonmalignant PET/CT findings: An expedited systematic review. Semin. Nucl. Med..

[81] Wang J., Huo L., Lin L., Niu N., Li X. (2023). In Vivo Fibroblast Activation of Systemic Sarcoidosis: A 68Ga-FAPI-04 PET/CT Imaging Study. Diagnostics.

[82] Zhang Z., Hou W., Pan G., Zuo C., Cheng C. (2024). Elevated 68 Ga-FAPI-04 Activity Due to Staphylococcus aureus Intracranial Infection. Clin. Nucl. Med..

[83] Malmberg C., Ripa R.S., Johnbeck C.B., Knigge U., Langer S.W., Mortensen J., Oturai P., Loft A., Hag A.M., Kjær A. (2015). 64Cu-DOTATATE for Noninvasive Assessment of Atherosclerosis in Large Arteries and Its Correlation with Risk Factors: Head-to-Head Comparison with 68Ga-DOTATOC in 60 Patients. J. Nucl. Med..

[84] Anzola L.K., Rivera J.N., Ramirez J.C., Signore A., Mut F. (2021). Molecular Imaging of Vulnerable Coronary Plaque with Radiolabeled Somatostatin Receptors (SSTR). J. Clin. Med..

[85] Lee H., Schubert E.K., Vidula M.K., Pryma D.A., Marchlinski F.E., Goldberg L.R., Clancy C.B., Rossman M.D., DiCarli M.F., Bravo P.E. (2023). Potential clinical utility of 68Ga-DOTATATE PET/CT for detection and response assessment in cardiac sarcoidosis. J. Nucl. Cardiol..

[86] Bobbio E., Dudás A., Bergström A., Esposito D., Angerås O., Taha A., van Essen M., Björkenstam M., Karason K., Bollano E. (2022). Incidental cardiac findings on somatostatin receptor PET/CT: What do they indicate and are they of clinical relevance?. J. Nucl. Cardiol..

[87] Jaleel J., Patel C.D., Chandra K.B., Ramakrishnan S., Seth S. (2023). Imaging Acute Myocarditis with 68Ga-DOTANOC PET/CT. Indian J. Nucl. Med..

[88] Boughdad S., Latifyan S., Fenwick C., Bouchaab H., Suffiotti M., Moslehi J.J., Salem J.E., Schaefer N., Nicod-Lalonde M., Costes J. (2021). 68Ga-DOTATOC PET/CT to detect immune checkpoint inhibitor-related myocarditis. J. Immunother. Cancer.

[89] Ćorović A., Wall C., Nus M., Gopalan D., Huang Y., Imaz M., Zulcinski M., Peverelli M., Uryga A., Lambert J. (2023). Somatostatin Receptor PET/MR Imaging of Inflammation in Patients With Large Vessel Vasculitis and Atherosclerosis. J. Am. Coll. Cardiol..

[90] Vorster M. (2023). Gallium-68 Labelled Radiopharmaceuticals for Imaging Inflammatory Disorders. Semin. Nucl. Med..

[91] Naftalin C.M., Leek F., Hallinan J.T.P.D., Khor L.K., Totman J.J., Wang J., Wang Y.T., Paton N.I. (2020). Comparison of 68Ga-DOTANOC with 18F-FDG using PET/MRI imaging in patients with pulmonary tuberculosis. Sci. Rep..

[92] Craig A., Kogler J., Laube M., Ullrich M., Donat C.K., Wodtke R., Kopka K., Stadlbauer S. (2023). Preparation of 18F-Labeled Tracers Targeting Fibroblast Activation Protein via Sulfur [18F]Fluoride Exchange Reaction. Pharmaceutics.

[93] Bhattacharya A., Kochhar R., Sharma S., Ray P., Kalra N., Khandelwal N., Mittal B.R. (2014). PET/CT with 18F-FDG-labeled autologous leukocytes for the diagnosis of infected fluid collections in acute pancreatitis. J. Nucl. Med..

[94] Meyer M., Testart N., Jreige M., Kamani C., Moshebah M., Muoio B., Nicod-Lalonde M., Schaefer N., Giovanella L., Prior J.O. (2019). Diagnostic Performance of PET or PET/CT Using 18F-FDG Labeled White Blood Cells in Infectious Diseases: A Systematic Review and a Bivariate Meta-Analysis. Diagnostics.

[95] Rastogi A., Bhattacharya A., Prakash M., Sharma S., Mittal B.R., Khandelwal N., Bhansali A. (2016). Utility of PET/CT with fluorine-18-fluorodeoxyglucose-labeled autologous leukocytes for diagnosing diabetic foot osteomyelitis in patients with Charcot’s neuroarthropathy. Nucl. Med. Commun..

[96] Aksoy S.Y., Asa S., Ozhan M., Ocak M., Sager M.S., Erkan M.E., Halac M., Kabasakal L., Sönmezoglu K., Kanmaz B. (2014). FDG and FDG-labelled leucocyte PET/CT in the imaging of prosthetic joint infection. Eur. J. Nucl. Med. Mol. Imaging.

[97] Yilmaz S., Vatankulu B., Ekmekciogu O., Sager S., Halac M. (2014). FDG and FDG-labelled leucocyte PET/CT in the imaging of prosthetic joint infection: Response to Lazzeri et al. Eur. J. Nucl. Med. Mol. Imaging.

[98] Krishnaraju V.S., Singh H., Kumar R., Sharma S., Mittal B.R., Bhattacharya A. (2021). Infection imaging using [18F]FDG-labelled white blood cell positron emission tomography-computed tomography. Br. J. Radiol..

[99] Sorlin A.M., López-Álvarez M., Rabbitt S.J., Alanizi A.A., Shuere R., Bobba K.N., Blecha J., Sakhamuri S., Evans M.J., Bayles K.W. (2023). Chemoenzymatic Syntheses of Fluorine-18-Labeled Disaccharides from [18F] FDG Yield Potent Sensors of Living Bacteria In Vivo. J. Am. Chem. Soc..

[100] Furumoto S., Shinbo R., Iwata R., Ishikawa Y., Yanai K., Yoshioka T., Fukuda H. (2013). In vitro and in vivo characterization of 2-deoxy-2-18F-fluoro-D-mannose as a tumor-imaging agent for PET. J. Nucl. Med..

[101] Li J., Zheng H., Fodah R., Warawa J.M., Ng C.K. (2018). Validation of 2-18F-Fluorodeoxysorbitol as a Potential Radiopharmaceutical for Imaging Bacterial Infection in the Lung. J. Nucl. Med..

[102] Rua M., Simón J.A., Collantes M., Ecay M., Leiva J., Carmona-Torre F., Ramos R., Pareja F., Pulagam K.R., Llop J. (2023). Infection-specific PET imaging with 18F-fluorodeoxysorbitol and 2-[18F]F-ρ-aminobenzoic acid: An extended diagnostic tool for bacterial and fungal diseases. Front. Microbiol..

[103] Zhu W., Yao S., Xing H., Zhang H., Tai Y.C., Zhang Y., Liu Y., Ma Y., Wu C., Wang H. (2016). Biodistribution and Radiation Dosimetry of the Enterobacteriaceae-Specific Imaging Probe [(18)F]Fluorodeoxysorbitol Determined by PET/CT in Healthy Human Volunteers. Mol. Imaging Biol..

[104] Singh S.B., Bhandari S., Siwakoti S., Bhatta R., Raynor W.Y., Werner T.J., Alavi A., Hess S., Revheim M. (2023). Is Imaging Bacteria with PET a Realistic Option or an Illusion?. Diagnostics.

[105] Hess S., Alavi A., Werner T., Høilund-Carlsen P.F. (2018). Molecular Imaging of Bacteria in Patients Is an Attractive Fata Morgana, Not a Realistic Option. J. Nucl. Med..

[106] Zaindi H., Alavi A. (2007). Current Trends in PET and Combined (PET/CT and PET/MR) Systems Design. PET Clin..

[107] Morrison L., Zembower T.R. (2020). Antimicrobial Resistance. Gastrointest. Endosc. Clin. N. Am..

[108] Giraudo C., Evangelista L., Fraia A.S., Lupi A., Quaia E., Cecchin D., Casali M. (2020). Molecular Imaging of Pulmonary Inflammation and Infection. Int. J. Mol. Sci..

[109] Bioletto F., Barale M., Parasiliti-Caprino M., Prencipe N., Berton A.M., Procopio M., Deandreis D., Ghigo E. (2021). Comparison of the diagnostic accuracy of 18F-Fluorocholine PET and 11C-Methionine PET for parathyroid localization in primary hyperparathyroidism: A systematic review and meta-analysis. Eur. J. Endocrinol..

[110] Braune A., Oehme L., Freudenberg R., Hofheinz F., van den Hoff J., Kotzerke J., Hoberück S. (2022). Comparison of image quality and spatial resolution between 18F, 68Ga, and 64Cu phantom measurements using a digital Biograph Vision PET/CT. EJNMMI Phys..

[111] Calabria F., Gallo G., Schillaci O., Cascini G.L. (2015). Bio-Distribution, Imaging Protocols and Diagnostic Accuracy of PET with Tracers of Lipogenesis in Imaging Prostate Cancer: A Comparison between 11C-Choline, 18FFluoroethylcholine and 18F-Methylcholine. Curr. Pharm. Des..

[112] Sanchez S., Currie G.M. (2020). Topical Sensor for the Assessment of Injection Quality for 18F-FDG, 68Ga-PSMA and 68Ga-DOTATATE Positron Emission Tomography. J. Med. Imaging Radiat. Sci..

[113] Yoon J.K., Park B.N., Ryu E.K., An Y.S., Lee S.J. (2020). Current Perspectives on 89Zr-PET Imaging. Int. J. Mol. Sci..

[114] Pickett J.E., Thompson J.M., Sadowska A., Tkaczyk C., Sellman B.R., Minola A., Corti D., Lanzavecchia A., Miller L.S., Thorek D.L. (2018). Molecularly specific detection of bacterial lipoteichoic acid for diagnosis of prosthetic joint infection of the bone. Bone Res..

[115] Kirienko M., Erba P.A., Chiti A., Sollini M. (2023). Hybrid PET/MRI in Infection and Inflammation: An Update About the Latest Available Literature Evidence. Semin. Nucl. Med..

[116] Rohan T., Hložanka P., Dostál M., Macek T., Fojtík Z., Šprláková-Puková A., Keřkovský M. (2024). Significance of F-18 FDG PET/MRI in the search for the etiology of inflammation of unclear origin and fever of unknown origin. Eur. J. Radiol..

[117] Tisseraud M., Goutal S., Bonasera T., Goislard M., Desjardins D., Le Grand R., Parry C.M., Tournier N., Kuhnast B., Caillé F. (2022). Isotopic Radiolabeling of the Antiretroviral Drug [18F]Dolutegravir for Pharmacokinetic PET Imaging. Pharmaceuticals.

[118] Manzarbeitia-Arroba B., Ruiz-Solis S., Vega-Perez D., Garcia-Alonso P., Tabuenca M.J. (2023). F-FDG PET/CT Findings in Monkeypox Infection in an HIV Patient. Clin. Nucl. Med..

